# The QCML dataset, Quantum chemistry reference data from 33.5M DFT and 14.7B semi-empirical calculations

**DOI:** 10.1038/s41597-025-04720-7

**Published:** 2025-03-08

**Authors:** Stefan Ganscha, Oliver T. Unke, Daniel Ahlin, Hartmut Maennel, Sergii Kashubin, Klaus-Robert Müller

**Affiliations:** 1 Google DeepMind, Zürich, Switzerland; 2 Google DeepMind, Berlin, Germany; 3 Machine Learning Group, TU Berlin and BIFOLD, Berlin, Germany; 4 Department of Artificial Intelligence, Korea University, Seoul, Korea; 5 Max Planck Institute for Informatics, Saarbrücken, Germany

**Keywords:** Cheminformatics, Scientific data

## Abstract

Machine learning (ML) methods enable prediction of the properties of chemical structures without computationally expensive *ab initio* calculations. The quality of such predictions depends on the reference data that was used to train the model. In this work, we introduce the QCML dataset: A comprehensive dataset for training ML models for quantum chemistry. The QCML dataset systematically covers chemical space with small molecules consisting of up to 8 heavy atoms and includes elements from a large fraction of the periodic table, as well as different electronic states. Starting from chemical graphs, conformer search and normal mode sampling are used to generate both equilibrium and off-equilibrium 3D structures, for which various properties are calculated with semi-empirical methods (14.7 billion entries) and density functional theory (33.5 million entries). The covered properties include energies, forces, multipole moments, and other quantities, e.g., Kohn-Sham matrices. We provide a first demonstration of the utility of our dataset by training ML-based force fields on the data and applying them to run molecular dynamics simulations.

## Background & Summary

Over the last two decades, machine learning (ML) methods have developed to become a standard tool in the field of computational chemistry. They allow to directly predict the properties of chemical structures without the need for solving the Schrödinger equation explicitly. Compared to *ab initio* calculations, which typically have high computational costs and scale poorly with system size, ML methods enable orders of magnitude speedup^1–4^. They have been used to construct machine-learned force fields (MLFFs)^5^ for materials^6^, small molecules^7–12^, and more recently, also larger systems with hundreds of atoms^13^ or proteins in solution^14^. Other applications range from accelerating molecular simulations^15^, for example by constructing Markov models^16^ or directly sampling equilibrium states^17^, over predicting wavefunctions^18,19^, to general exploration of chemical space^20,21^, for example to discover novel materials^22^ or to solve inverse chemical design tasks^23^.

What most of these applications have in common is that they require high quality reference properties obtained from conventional quantum chemistry methods, such as density functional theory (DFT)^24^, for training the ML models^25^. To generate such data, large databases of chemical compounds are often used as starting point. For example, PubChem^26^ and ChEMBL^27^ provide an extensive catalogue of experimentally observed molecules and known bioactive compounds, respectively. Other databases, such as GDB-11^28,29^, GDB-13^30^, and GDB-17^31^ take a different approach and systematically enumerate a subspace of all possible chemical compounds, thus also including molecules which may never have been synthesised. By sampling compounds from different subsets of chemical space and computing their properties with first-principles methods, data for training ML models for different purposes can be generated.

Over the years, many such collections of *ab initio* data have emerged. Among the first databases for the development and benchmarking of ML models were QM7^32,33^ and QM9^34^. They contain properties such as atomisation energies, dipole moments, and HOMO/LUMO energies for equilibrium structures of 7,165 and 133,885 molecules sampled from GDB-13^30^ and GDB-17^31^, respectively, and are most suitable for training ML models for chemical space exploration. The PubchemQC project^35–38^ has released multiple datasets since 2015, with the latest version, B3LYP/6-31G*//PM6^38^ covering equilibrium structures of 86M molecules corresponding to 93.7% of PubChem. Other datasets also include off-equilibrium structures, for example, ANI-1^39^ consists of energies and forces for more than 20 million conformations of around 60 thousand organic molecules and QM7x^40^ contains data for more than 4 million conformations of the molecules in QM7. Datasets such as QMugs^41^ on the other hand focus on drug discovery and contain both semi-empirical and DFT data for about 2 million equilibrium structures of 665 thousand molecules with up to 100 heavy atoms sampled from ChEMBL^27^. Other efforts, such as SPICE^42^, focus on modelling the interaction between small molecules and proteins.

While a plethora of *ab initio* datasets already exist – each covering different classes of compounds, elements, and molecular properties – they are typically only suitable for training specific types of ML models. For example, databases containing only equilibrium structures may be sufficient for chemical space exploration, but cannot be used to train MLFFs, which also require reference data for off-equilibrium conformations. Similarly, datasets that only include structures with certain elements, e.g., H, C, N, and O atoms, cannot be used to train ML models for predicting the properties of compounds that contain heavier elements. Unfortunately, combining information from different sources is not straightforward, because structures are usually sampled with inconsistent schemes and properties are computed at different levels of theory. A dataset that covers all elements, all of chemical (and conformational) space, and contains a multitude of properties would enable the training of *foundation models*, which are broadly applicable over chemical space and different downstream tasks.

In this work, we take a first step towards the construction of such a *universal* database for quantum chemistry, based on the observation that ML models can often extrapolate to much larger structures, as long as all relevant local bonding patterns are covered in the training set^14,43^. Based on 17.2M chemical graphs constructed from fragments of known molecules and synthetically generated graphs, we sample 14678M different conformations (at temperatures between 0 and 1000 K) and calculate properties with semi-empirical methods. We then select a randomly chosen subset of 33.5M structures, for which we run DFT calculations. Our dataset, which we call QCML dataset^44^, offers a wide range of properties, including energies, forces, multipole moments, and matrix quantities (such as the Hamiltonian).

## Methods

**Fig. 1:**
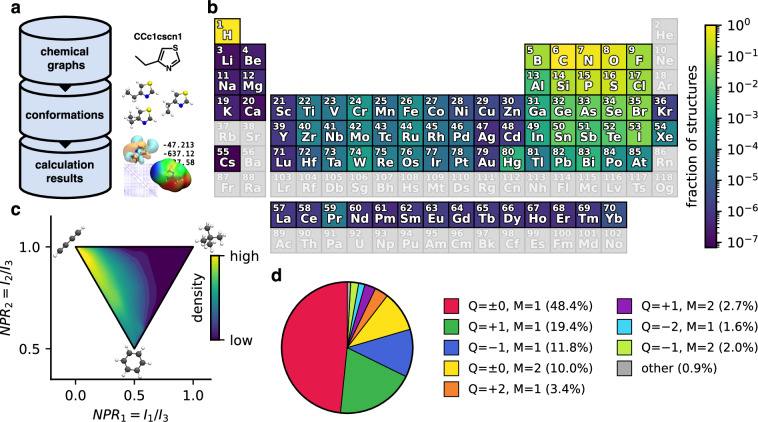
(**a**) Overview of the hierarchical organisation of the QCML dataset^44^. Each chemical graph is associated with multiple conformations (3D structures), and each conformation can have many corresponding calculation results (multiple properties calculated at different levels of theory). (**b**) Chemical diversity of structures in the QCML dataset across the periodic table. The colour indicates which fraction of structures contains a given element (greyed out entries are not contained in any structure). As expected, the majority of structures contains only H, C, N, O, S, and P atoms, however, nearly all elements with atomic number *Z* < 86 are represented in at least some structures. (**c**) Shape diversity of conformations in the QCML dataset expressed by NPRs (normalised principal moment of inertia ratios, *I*_*k*_ denotes the *k*-th principal moment of inertia)^86^. Structures within the QCML dataset with archetypal disc (0.5, 0.5), rod (0.0, 1.0), and sphere (1.0, 1.0) shapes are shown for reference. We note that while the QCML dataset contains conformations in all regions of the plot, entries are more densely concentrated near rod- and disc-like shapes (the density is shown on a logarithmic scale). (**d**) Distribution of electronic states in the QCML dataset, indicated by their total charge (Q) and multiplicity (M). While the majority of structures are in a neutral singlet state (Q = 0, M = 1), the QCML dataset also contains many structures with a non-zero total charge (Q ≠ 0) and even doublet (M = 2) states.

The QCML dataset^44^ consists of three types of data organised in a hierarchical manner, with a record of chemical graphs at the top of the hierarchy, followed by conformations (3D structures) in the middle, and the results of quantum chemical calculations at the bottom (see Fig. 1a). It contains structures with elements covering a large fraction of the periodic table (see Fig. 1b), diverse molecular shapes (see Fig. 1c), and various electronic states (see Fig. 1d). There is a one-to-many relationship between entries at a given level of the database hierarchy and those in the next lower level. For example, the same chemical graph may have multiple conformations as children, but each calculation result has exactly one conformation as parent. This hierarchical organisation allows straightforward searching and filtering of the data (e.g., to select all data that belongs to a specific molecule). Further, it enables data generation in a largely automated manner while ensuring high data quality through several automated checks and filters (see Technical Validation for details).

In the following, the three types of data and how they are generated are described in more detail.

### Chemical Graphs

Chemical graphs are a representation of the structural formula of a chemical compound. They are undirected graphs, where every vertex is labelled with a specific element (e.g., H, C, N, O, …) and edges are labelled with the kind of chemical bond they represent (e.g., single, double, aromatic, …). We store chemical graphs as strings using the SMILES^45^ (simplified molecular-input line-entry system) notation. A single chemical graph can typically be represented by multiple different valid SMILES strings. However, we require that all representations in our database are unique, so that redundant entries (duplicate chemical graphs) can be filtered out. To achieve this, we use Open Babel 3.1.1^46^ (http://openbabel.org) to generate a (unique) canonical^47^ SMILES representation for each chemical graph. In addition to atom connectivity, SMILES strings may encode information about formal charges and even the configuration of a compound, e.g., the parity of a stereogenic carbon atom, or whether a double bond is in *E* or *Z* configuration (2.5D representation). While this information is typically considered optional, we require that all SMILES representations in our database are unambiguous (i.e., cannot stand for multiple configurational isomers/stereoisomers).

**Fig. 2:**
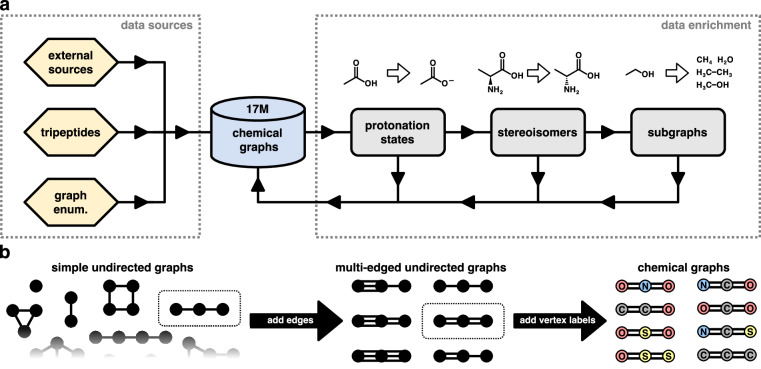
(**a**) Overview of the workflow used for the creation of the chemical graphs database. Chemical graphs are imported from external data sources (e.g., PubChem^26^) or created from scratch (see text for details). The graphs are then fed through a data enrichment pipeline whose outputs are added back to the database. (**b**) Schematic depiction of the graph enumeration procedure for systematically generating chemical graphs (hydrogen atoms are implicit and therefore not shown). For better clarity, we only show a small fraction of the possible simple undirected graphs and subsequent steps (adding edges and vertex labels) are only visualised for specific graphs (indicated by the dotted rectangles).

Our goal is to build a database of small chemical graphs that cover the chemical space of possible molecules up to a specified number of heavy (non-hydrogen) atoms as completely as possible. To achieve this, we source chemical graphs of known molecules from multiple existing databases. For instance, molecules up to 50 heavy atoms from PubChem, see Data sources. Additionally, we generate small chemical graphs systematically. Afterwards, all imported chemical graphs go through data enrichment steps that generate related chemical graphs (e.g., subgraphs, stereoisomers, etc), which are re-imported into our database. Finally, we keep all chemical graphs with eight heavy atoms or less and use them as the starting point for subsequent steps in the data generation pipeline (see Fig. 2a for an overview of the general workflow and below for details on the individual steps). We note that while chemical graphs may be present in multiple data sources (and/or generated by multiple enrichment steps), this is inconsequential, because duplicate chemical graphs can be easily filtered out based on their canonical SMILES representation. Also, while not exactly duplicates, for chemical graphs which are mirror images of each other (enantiomers), we arrange their canonical SMILES in lexicographic order and skip the second enantiomer in subsequent steps of our pipeline (generating 3D structures and running quantum chemical calculations). Since the properties of a molecule either do not change under reflections at all, or via a trivial transformation, skipping one of the enantiomers prevents the generation of redundant entries in the database (most recent ML models for quantum chemistry respect physical symmetry relations by construction)^5^.

#### Data sources

##### External sources

We import chemical graphs (represented as SMILES strings) from GDB-11^28,29^, GDB-13^30^, and GDB-17’s^31^ “50 million” set (https://gdb.unibe.ch/downloads/), as well as all molecules from PubChem^26^ with less than or equal to 50 heavy atoms (https://ftp.ncbi.nlm.nih.gov/pubchem/Compound). In total, imports from external sources amount to roughly one billion unique SMILES.

##### Tripeptides

Due to the biological importance of proteins, we want to make sure that chemical graphs in our database cover substructures that can occur in proteins. Previous work found that tripeptides connected by covalent bonds (peptide bonds and/or disulfide bridges) already contain *all* substructures that can occur in natural proteins (without post-translational modifications) with up to (at least) eight heavy atoms^9^. We consider all 22 known proteinogenic amino acids (including selenocysteine and pyrrolysine)^48^ and generate tripeptides as follows: First, we enumerate all 22^3^ = 10 648 combinations of three amino acids. Then, for peptides containing at least two cysteine residues, we create additional variants containing disulfide bridges (for tricysteine, all possible ways of forming disulfide bridges are considered). Further variants are generated by splitting any of the existing peptide bonds, but keeping only structures that are still fully connected by covalent bonds (via disulfide bridges). This procedure leads to 154 additional structures for a total of 10 802 peptides. Finally, the generated peptides are converted to SMILES representations and imported into the chemical graphs database. Since our data enrichment pipeline includes a subgraph generation step (see below), smaller peptides or substructures do not need to be created explicitly, because they are introduced into the database at a later step anyway.

##### Graph enumeration

To reduce potential “holes” in our covering of chemical space – molecules which are neither present in other data sources (see above), nor generated in subsequent data enrichment steps (see below) – we systematically generate chemical graphs and import them into our database. Although it is infeasible to enumerate every possible chemical graph (and thus cover chemical space completely) due to the associated combinatorial explosion, by limiting the total number of heavy atoms and allowed elements, it is still possible to find molecules with unusual bonding patterns, which would otherwise be missed. We first create all possible (connected) simple undirected graphs consisting of at most five vertices using the nauty-geng program^49,50^. Then, we generate additional graphs by introducing between zero and two new edges (connecting the same two vertices) for each existing edge, so that the number of edges encodes the bond order (i.e., single, double, or triple bonds) – as long as additional edges do not increase the degree of any vertex beyond six (all possible combinations of multi-edges that meet this criterion are considered). Finally, we label vertices with elements chosen from the “organic subset” (B, C, N, O, P, S, F, Cl, Br, and I)^45^, making sure that the vertex degree does not exceed the maximum valency of the selected element (see Supplementary Table 3). Graphs with all possible combinations of labels that meet this requirement are created. Importantly, for elements in the organic subset, the SMILES notation does not require explicitly specifying hydrogen atoms (additional bonds to hydrogen atoms are implicit, see Supplementary Table 3). The full graph enumeration procedure is summarised in Fig. 2b.

#### Data enrichment

To increase the versatility of the chemical graphs included in our database, we enrich them using chemical knowledge as described below.

##### Protonation states

Many chemical graphs contain substructures such as carboxyl or amine groups, which can exist in different protonation states. Arguably, (de)protonation is one of the most important chemical reactions and a fundamental step in many catalytic processes. Thus, to increase the diversity of chemical graphs in our database and include structures important for the description of (de)protonation reactions, we generate additional chemical graphs from a “seed graph” as follows: First, we use the built-in pH model of Open Babel to identify all sites where (de)protonation can occur and record the possible protonation states for each site. Then, we enumerate all possible combinations of states for the different protonation sites (even if they would not typically occur at the same pH level) and add them to the chemical graphs database. For example, for the input graph CC(=O)O (acetic acid), we would generate the variant [O-]C(=O)C (acetate), see Fig. 2a.

##### Stereoisomers

As mentioned above, we require that all chemical graphs in our database have a fully specified stereochemistry, i.e., we disallow graphs such as CC=CC (but-2-ene) and instead only allow graphs such as C/C=C/C ((*E*)-but-2-ene) or C/C=C\C ((*Z*)-but-2-ene). To ensure that our database contains all possible stereoisomers of a given chemical graph, we first use Open Babel to identify all tetrahedral atoms and bonds with cis/trans isomery. Then, we generate new chemical graphs with all possible combinations of winding orders (for tetrahedral atoms) and cis/trans orientations (for double bonds). For example, for an input such as C[C@@H](C(=O)O)N (L-alanine), we would also generate C[C@H](C(=O)O)N (D-alanine), see Fig. 2a. We note that this procedure may create different SMILES strings encoding the same molecule, for example C/C=C/C and C\C=C\C, which both represent (*E*)-but-2-ene. However, this is of no concern, because (as mentioned above) all SMILES strings undergo canonisation before they are added to our database, thus duplicates can be easily detected and filtered out by string comparison.

##### Subgraphs

Following Ref. ^43^, we generate subgraphs (also referred to as “amons”^43^) of a chemical graph by first recording the valency of each heavy atom. Then, (explicit) hydrogen atoms are removed and all connected subgraphs (now consisting only of heavy atoms) are generated. Finally, hydrogen atoms are added back to the subgraphs until all heavy atoms reach the valency they had in the original “seed graph”. For example, for the input CCO (ethanol), we generate the subgraphs CC (ethane), CO (methanol), C (methane), and O (water), see Fig. 2a.

##### Restriction to eight heavy atoms

We apply above data imports and enrichment for input graphs of all mentioned lengths, but restrict the downstream processing to graphs with maximal eight heavy atoms. For instance, for a chemical graph from PubChem consisting of 50 heavy atoms all possible (unique) subgraphs of lengths up to and including 8 heavy atoms are processed further.

### Conformations (3D structures)

Although chemical graphs are a convenient way to categorise molecules purely based on atom connectivity, they are insufficient to perform quantum chemical calculations: For this purpose, the exact spatial arrangement of atoms (3D structure), as well as the electronic state, are required. To assign the latter, we derive the total charge from the SMILES representation of the chemical graph (by summing all formal charges) and assume the lowest number of unpaired electrons consistent with the total charge, i.e., we assign singlet (resp. doublet) states for an even (resp. odd) number of electrons. Assigning positions to individual atoms is less straightforward, because infinitely many arrangements are possible. Moreover, it is not even necessarily clear which chemical graph to assign to a particular spatial arrangement of atoms, since the conformational spaces of isomeric structures smoothly transition into each other. Uniformly sampling from all possible arrangements is both practically infeasible and not meaningful, because most of the sampled conformations would correspond to “unphysical” structures associated with large potential energies, which would not (or only very rarely) occur in nature.

**Fig. 3:**
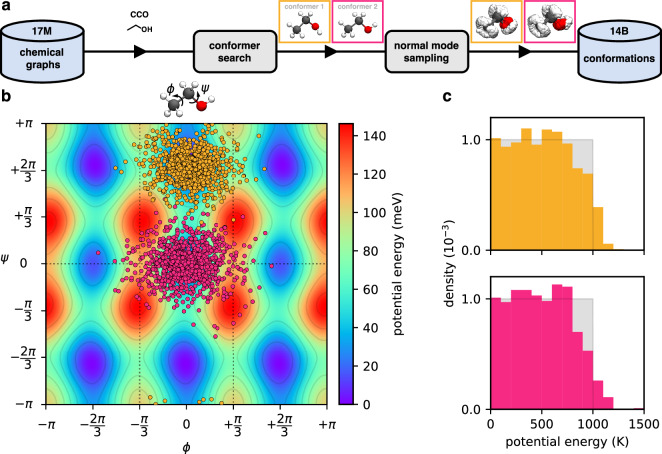
(**a**) Overview of the workflow used for creating the database of conformations from chemical graphs; CCO (ethanol) is used as an example. First, a conformer search is performed to find (local) minima on the potential energy surface (PES). Then, multiple off-equilibrium structures are generated via normal mode sampling for each found conformer. (**b**) Projection of normal mode samples for the two conformers (yellow: conformer 1, pink: conformer 2) of ethanol onto a two-dimensional cut of the PES along rotations of the CH_3_ (*ϕ* angle) and OH (*ψ* angle) groups (energies are given w.r.t. the global minimum, conformer 1). The dotted lines indicate regions of the PES which are equivalent under symmetry operations. (**c**) Histogram of sampled potential energies w.r.t. the corresponding minimum for off-equilibrium structures of the two unique conformers of ethanol (top: conformer 1, bottom: conformer 2). Here, the energy *E* is measured in Kelvin, referring to the corresponding temperature $T=\frac{2E}{{k}_{B}{n}_{f}}$, where *k*_*B*_ is the Boltzmann constant and *n*_*f*_ denotes the number of internal degrees of freedom (*n*_*f*_ = 12 for ethanol). For reference, uniform distributions between 0 K and 1000 K are shown in grey.

We aim to build a database of 3D structures that contains mainly “physical” conformations (that would occur naturally on reasonably small energy scales). At the same time, the sampled structures should be as diverse as possible to maximise information gain when running quantum mechanical calculations for different conformations of the same chemical graph. To achieve this, we follow a similar approach as previous work^40^ and first search for *conformers* – local minima of the potential energy surface (PES) – and then distort them in a “chemically meaningful way” to sample diverse off-equilibrium conformations (see Fig. 3a for an overview of the general workflow and below for details on the individual steps).

#### Conformer Search

Starting from a chemical graph, we generate a first guess for a 3D structure using the OBBuilder class included in Open Babel, which assigns coordinates to atoms using simple empirical rules based on their connectivity. Since structures generated in this way may contain “unphysical artefacts” (e.g., partially overlapping groups of atoms), they are pre-optimised with the conjugate gradients methods^51^ using the universal force field (UFF)^52^ implementation included in Open Babel, until the energy between subsequent steps does not change by more than 1 J mol^−1^ atom^−1^ (or a maximum of 100,000 steps is reached). We then re-optimise the structure at the GFN0-xTB^53^ level of theory with the BFGS (Broyden-Fletcher-Goldfarb-Shanno) algorithm^54–58^ implemented in ASE (Atomic Simulation Environment)^59^ until the maximum force acting on any atom is smaller than 5 meV Å^−1^. For many inputs, the pre-optimisation step (using the UFF) is redundant, but it typically speeds up the convergence of the optimisation with GFN0-xTB and can be crucial in cases where the initial structure contains artefacts (e.g., partially overlapping atoms).

After an optimised structure is obtained, we use the OBConformerSearch class included in Open Babel to search for other conformational isomers (conformers). The conformer search is based on a genetic algorithm^46^ that considers different combinations of substructure orientations around “rotatable bonds”. By default, Open Babel excludes some bonds in this search (even though they can be rotated), but we explicitly also include single bonds that are part of a ring structure (for all but three-membered rings), or connect to a heavy atom that is only bound to hydrogen atoms (e.g., R–OH). We use Open Babel’s OBRMSDConformerScore scoring function to determine the “fitness” of candidates in the population (encouraging a structurally diverse set of conformers) and set the number of conformers considered during the search to 10 (all other hyperparameters are kept at their default values). After the conformer search is finished, we optimise all newly found structures as described above (pre-optimisation with UFF followed by optimisation with GFN0-xTB). Any “duplicate” conformers (related by symmetry) are filtered out based on the (symmetry-aware) root-mean-square deviation (RMSD) calculated with the OBAlign class (conformers with a RMSD < 0.05 Å to another structure are considered duplicates). Importantly, we also filter out mirror image conformers in this step, following the same reasoning as for filtering out enantiomers at the chemical graph stage (see above). For example, for a molecule such as CCO (ethanol), only two “unique” conformers exist (see Fig. 3a); all other conformers are related to one of these by trivial symmetry relations (e.g., rotation around single bonds, permutation of equivalent atoms, or reflections).

#### Normal Mode Sampling

Starting from the conformers found in the previous step, we generate between 100 and 1,000 off-equilibrium structures (depending on the number of heavy atoms, see Supplementary Table 4) using a variant of normal mode sampling^60^. The basic idea is to perform a normal mode analysis^61^, which implicitly approximates the chemical system as a collection of uncoupled harmonic oscillators that independently move along orthogonal directions (the normal modes), so that the overall motion of the system can be described by their superposition. Thus, by randomly distributing energy into different modes, it is possible to sample uncorrelated conformations “around” an equilibrium structure with a specific (relative) potential energy. Note that the implicit harmonic approximation used during normal mode sampling is only meaningful at stationary points of the PES, where first-order contributions vanish (the forces are zero), which we ensure when generating the conformers in the previous step (see above).

More precisely, for a structure of *N* atoms, we calculate the 3*N* × 3*N* (mass-weighted) Hessian **H** (in Cartesian coordinates) using finite differences of the GFN0-xTB forces with a standard six-point stencil^62^ and a displacement of 10^−3^ Å. At this stage, we check that the energy increases for all displacements used for evaluating the six-point stencil and discard conformers that do not meet this criterion. Diagonalisation of **H** then gives the normal modes {**q**_1_, …, **q**_3*N*_} (related to the eigenvectors) and associated force constants {*k*_1_, …, *k*_3*N*_} (related to the eigenvalues) (see Ref. ^61^ for a detailed description of normal mode analysis). We discard the six (resp. five for linear structures) normal modes associated with rigid translations and rotations, leaving only the *n*_*f*_ = 3*N* − 6 (resp. *n*_*f*_ = 3*N* − 5) internal degrees of freedom (DOFs). According to the equipartition theorem, on average, the energy in a specific DOF is $\frac{1}{2}{k}_{B}T$ at temperature *T* (*k*_*B*_ is the Boltzmann constant)^63^. Thus, to account for varying numbers of DOFs between structures of different sizes, we do not directly sample energies, but instead select a temperature *T* uniformly between 0 K and 1000 K and set the energy to $E=\frac{1}{2}{n}_{f}{k}_{B}T$. This energy is then distributed over the normal modes according to

1 $${E}_{i}=\frac{{w}_{i}^{2}}{{\sum }_{i=1}^{{n}_{f}}{w}_{i}^{2}}E\,,$$

where the raw weights *w*_*i*_ are drawn from a standard normal distribution. The magnitude of the displacement *δ*_*i*_ from the equilibrium position along the normal mode *i* can then be derived from the energy of the corresponding harmonic oscillator

2 $${E}_{i}=\frac{1}{2}{k}_{i}{\delta }_{i}^{2}\quad \Rightarrow \quad {\delta }_{i}=\pm \sqrt{\frac{2{E}_{i}}{{k}_{i}}}\,,$$

where we set the sign of *δ*_*i*_ to ${\rm{sgn}}\,{w}_{i}$ and the *k*_*i*_ correspond to the force constants obtained from normal mode analysis (see above). An off-equilibrium conformation is then generated by adding the linear combination of all displacements

3 $$\mathop{\sum }\limits_{i=1}^{{n}_{f}}{\delta }_{i}{{\bf{q}}}_{i}$$

to the equilibrium positions. In practice, the assumption of harmonic behaviour underlying normal mode analysis is only valid for small *δ*_*i*_. Particularly, when large displacements are expressed in Cartesian coordinates, they are typically a bad approximation for orthogonal directions into which molecular motions can be decomposed. We empirically find that a more “natural” coordinate system (in which the harmonic approximation stays valid for larger displacements) is given by the Z-matrix, which is an internal coordinate representation based on distances, angles, and dihedrals. Thus, we use the chemcoord^64^ package to transform equilibrium positions and normal modes to a Z-matrix representation before generating conformations with Eq. (3) (if the Z-matrix generation fails, e.g., for linear molecules, we fall back to Cartesian coordinates).

The rationale for drawing the energies of conformations uniformly is that it leads to an even distribution of samples on the PES (see Supplementary Figure 5 for a visualisation). Setting the energy according to temperatures between 0 K and 1000 K is typically enough to also sample the transition regions connecting the basins of attraction of different conformers on the PES (see Fig. 3b). However, because the PES of real molecules only approximately behaves as a system of independent harmonic oscillators (even when Z-matrix coordinates are used), the actual energy distribution of sampled conformations deviates slightly from uniformity, especially for large energies/displacements (see Fig. 3c). Unrelated to this effect, we note that the distributions of samples in Fig. 3b appear to be more densely concentrated close to the minima compared to what is shown in Supplementary Figure 5, but this is only a visual artefact: Because Fig. 3b shows a two-dimensional cut of a twelve-dimensional PES, displacements in directions orthogonal to the cut appear “squashed” in the projection.

### Quantum Chemical Calculations

Next, we run quantum chemical calculations and compute properties using the 3D structures generated in the previous step of the data generation pipeline as input. We compute reference data with semi-empirical tight-binding methods for all conformations and at the density functional theory (DFT)^24^ level of theory for a randomly selected subset. Finally, since the accurate description of dispersion interactions is a well-known weakness of DFT^65^, we also compute dispersion corrections. Details on the individual steps are given below.

#### Semi-empirical Calculations

We compute energy, forces, Wiberg/Mayer bond orders^66,67^, orbital occupations, and orbital energies using the GFN0-xTB^53^ and GFN2-xTB^68^ methods. We also save the partial charges derived from electronegativity equilibration (see Ref. ^53^ for details) used for evaluating the Hamiltonian (for GFN0-xTB) or Mulliken charges (for GFN2-xTB). All calculations use the default parameters for accuracy and electronic temperature.

#### Density Functional Theory Calculations

DFT calculations are performed with the FHI-aims^69^ software using the PBE0^70,71^ functional and the default tight settings for the basis set. Note that FHI-aims uses numeric atom-centred orbitals (NAOs) instead of Gaussian type orbitals (GTOs) as basis functions. The default tight settings include basis function up to and including *tier 2*, see Ref. ^69^ for details. For open-shell systems, we set the spin keyword to collinear and distribute the initial moment evenly across all atoms; closed-shell systems are calculated with a restricted ansatz (spin none). All calculations use the scalar-relativistic atomic ZORA correction by setting the keyword relativistic atomic_zora scalar. Convergence criteria for the self-consistent field (SCF) iterations are set to sc_accuracy_eev 1e-3, sc_accuracy_etot 1e-6, sc_accuracy_forces 1e-4, and sc_accuracy_rho 1e-5. Any calculations that do not meet the convergence criteria within 1,000 SCF iterations are discarded.

It is well-known that the accuracy of DFT functionals can vary drastically depending on which particular classes of chemical compounds they are applied to. Since our goal is to broadly cover the space of possible compounds, naturally, any choice of DFT functional will be suboptimal for some compounds. The PBE0 functional was chosen for its low degree of empiricism and because it performs well in DFT benchmarks^72^ and has been shown to agree with high-level quantum chemistry methods and experiment for, e.g., polypeptides^73,74^, supramolecular complexes^75^, and molecular crystals^76^, especially when combined with dispersion corrections (see below).

#### Dispersion Corrections

To calculate the MBD-NL^77^ dispersion correction, we use the built-in implementation of FHI-aims^69^ by setting the keyword many_body_dispersion_nl, which automatically selects appropriate default parameters for the given functional (in our case, PBE0). Additionally, we calculate the DFT-D4^78^ correction using the dftd4 software available from https://github.com/dftd4/dftd4 using the recommended default parameters for the PBE0 functional.

## Data Records

All data is available as Tensorflow datasets (TFDS)^79^ in the publicly accessible Google Cloud storage bucket gs://qcml-datasets/tfds/^44^. For details about accessing Google Cloud storage in general see for instance (https://cloud.google.com/storage/docs/downloading-objects). As a TFDS, the data is ready for model training, but can also by used for data analyses or converting to other formats.

**Table 1:** Number of different types of data per number of heavy atoms.

heavy atoms	chemical graphs	GFN0-xTB results	GFN2-xTB results	DFT results
0	1	1 000	1 000	1 000
1	370	369 990	361 478	326 467
2	2 334	2 389 991	2 372 510	2 250 214
3	10 523	13 271 805	13 226 729	1 305 872
4	48 118	95 019 955	94 841 248	9 450 335
5	198 981	629 014 462	628 004 953	6 262 708
6	810 712	1 811 130 555	1 808 485 469	1 806 514
7	3 237 710	3 658 668 095	3 653 561 990	3 649 109
8	12 927 258	8 468 298 315	8 456 933 897	8 443 952
*Total*	17 236 007	14 678 164 168	14 657 789 274	33 496 171

GFN0-xTB and GFN2-xTB calculations were performed for every available normal mode sample, DFT calculations for subsamples of fractions decreasing with the number of heavy atoms.

The data is organised following the default TFDS directory structure: dataset/config/version. Our dataset name is qcml. There are two main collections: 15B xTB GFN0 and GFN2 examples (config: xtb_all/), and 33M PBE0 results (configs dft_.../). For the latter, for instance, qcml/dft_pbe0_energy/1.0.0/ contains the data (shards) and metadata of PBE0’s energy in version 1.0.0. Feature names correspond to the ones listed in Table 2.

**Table 2:** Chemical properties in the QCML dataset^44^.

key	type	shape	unit	description
**inputs to quantum chemical calculations**				
charge	int	()	*e*	total charge
multiplicity	int	()	—	spin multiplicity
atomic_numbers	u8	(N)	—	atomic numbers (nuclear charges)
positions	f32	(N,3)	*a*_0_	atomic positions
**potential energy surface**				
{gfn0∣gfn2∣pbe0}_energy	f64	()	*E*_h_	energy
{gfn0∣gfn2∣pbe0}_forces	f32	(N,3)	${E}_{{\rm{h}}}{a}_{0}^{-1}$	forces
{gfn0∣gfn2∣pbe0}_formation_energy	f64	()	*E*_h_	formation energy, see Supplementary Section 1
pbe0_electronic_free_energy	f64	()	*E*_h_	FHI-aims electronic free energy
pbe0_zero_broadening_corrected_energy	f64	()	*E*_h_	FHI-aims zero broadening estimate of energy
**dispersion corrections**				
{mbd∣d4}_energy	f64	()	*E*_h_	dispersion energy
{mbd∣d4}_forces	f32	(N,3)	${E}_{{\rm{h}}}{a}_{0}^{-1}$	dispersion forces
{mbd∣d4}_c6_coefficients	f32	(N)	${E}_{{\rm{h}}}{a}_{0}^{6}$	atomic *C*_6_ coefficients
{mbd∣d4}_polarizabilities	f32	(N)	${a}_{0}^{3}$	atomic polarizabilities
**multipole moments** (see Supplementary Section 1 for details and conventions used)				
{gfn0∣gfn2∣pbe0}_dipole	f32	(3)	*ea*_0_	dipole moment
pbe0_quadrupole	f32	(5)	$e{a}_{0}^{2}$	quadrupole moment
pbe0_octupole	f32	(7)	$e{a}_{0}^{3}$	octupole moment
pbe0_hexadecapole	f32	(9)	$e{a}_{0}^{4}$	hexadecapole moment
**population analysis and partial charges**				
{gfn0∣gfn2}_wiberg_bond_orders	f32	(N,N)	—	matrix of Wiberg bond orders between atoms
gfn0_eeq_charges	f32	(N)	*e*	partial charges derived from EEQ scheme
{gfn2∣pbe0}_mulliken_charges	f32	(N)	*e*	partial charges from Mulliken population analysis
pbe0_mulliken_spins	f32	(N)	—	per-atom spin density from Mulliken population analysis
pbe0_loewdin_charges	f32	(N)	*e*	partial charges from Löwdin population analysis
pbe0_loewdin_spins	f32	(N)	—	per-atom spin density from Löwdin population analysis
pbe0_hirshfeld_charges	f32	(N)	*e*	partial charges from Hirshfeld population analysis
pbe0_hirshfeld_dipoles	f32	(N,3)	*ea*_0_	atomic dipoles from Hirshfeld population analysis
pbe0_hirshfeld_quadrupoles	f32	(N,5)	$e{a}_{0}^{2}$	atomic quadrupoles from Hirshfeld population analysis
pbe0_hirshfeld_spins	f32	(N)	—	per-atom spin density from Hirshfeld population analysis
pbe0_hirshfeld_volumes	f32	(N)	${a}_{0}^{3}$	atomic volumes *V* from Hirshfeld population analysis
pbe0_hirshfeld_volume_ratios	f32	(N)	—	*V*/*V*_free_, where *V*_free_ are Hirshfeld volumes of free atoms
d4_atomic_charges	f32	(N)	*e*	partial charges assigned by D4 method
**orbital information and matrix quantities** (see Supplementary Section 1 for details and conventions used)				
{gfn0∣gfn2∣pbe0}_orbital_energies_{a∣b}	f32	(B)	*E*_h_	orbital energies for *α*/*β* electrons
{gfn0∣gfn2∣pbe0}_orbital_occupations_{a∣b}	f32	(B)	—	orbital occupations for *α*/*β* electrons
pbe0_orbital_coefficients_{a∣b}	f64	(B,B)	—	orbital coefficients
pbe0_density_matrix_{a∣b}	f64	(B,B)	—	density matrix
pbe0_hamiltonian_matrix_{a∣b}	f64	(B,B)	*E*_h_	Hamiltonian (Kohn-Sham) matrix
pbe0_core_hamiltonian_matrix	f64	(B,B)	*E*_h_	core Hamiltonian matrix
pbe0_overlap_matrix	f64	(B,B)	—	overlap matrix (overlap integrals)
**properties related to electron density stored on numerical integration grid** (see Supplementary Section 1 for details and conventions used)				
pbe0_grid_points	f64	(M,3)	*a*_0_	positions of grid points
pbe0_grid_weight	f64	(M)	—	weights *w*_*i*_ of individual grid points
pbe0_grid_density_{a∣b}	f64	(M)	*e*	electron density *ρ*
pbe0_grid_density_gradient_{a∣b}	f64	(M,3)	$e{a}_{0}^{-1}$	gradient of the electron density ∇ *ρ*
pbe0_grid_density_laplacian_{a∣b}	f64	(M)	$e{a}_{0}^{-2}$	Laplacian of the electron density *Δρ*
pbe0_grid_kinetic_energy_density_{a∣b}	f64	(M)	*E*_h_	kinetic energy density

The placeholders N, B, and M in shape specifications vary between entries and refer to the number of atoms, basis functions, and points of the numerical integration grid, respectively. Please refer to Supplementary Section 1 in the Supplementary Information for more detailed descriptions of individual properties, documentation of additional metadata fields, and information about the conventions used in the QCML dataset.

## Technical Validation

The technical validation involved automated checks and filters on chemical graphs, conformations, and quantum chemical calculations to ensure data quality. Additionally, we analysed the distributions of formation energy, maximum force, minimum inter-atomic distance and bond order, and implemented a flag for potential outliers. On the resulting non-outlier data, we demonstrate how to train a state-of-the-art machine learning force field to chemical accuracy. Additionally, we observe a high degree of correlation of results between different levels of theory which indicates feasibility of transfer learning approaches starting from our 14.7B semi-empirical data points.

### Automated checks and filters

The large amounts of data processed for generating the QCML dataset^44^ prevent manual inspection of all generated entries. However, we still use several automated checks to individually check all data points and filter out entries that do not meet our criteria for high quality data.

#### Chemical Graphs

All chemical graphs that are imported into our database (either from external sources or via data enrichment steps) are sanitised by removing isotope information from SMILES strings. Further, all SMILES which contain the character ‘.’ (representing disconnected chemical graphs), or for which the OBBuilder class from Open Babel cannot assign an initial 3D structure, are filtered out. We only generate conformations for chemical graphs with at most 8 heavy atoms, see Table 1.

#### Conformations (3D structures)

All conformers for which the GFN0-xTB geometry optimisation (see Conformer Search) does not converge within 1,000 steps are discarded. Further, we use the Wiberg/Mayer bond orders^66,67^ computed with GFN0-xTB to automatically check for consistency with the parent chemical graph: If any bond order deviates by more than 0.5 from the value expected from the graph connectivity, the structure is filtered out (for single, aromatic, double, and triple bonds, the expected bond orders are 1, 1.5, 2, and 3, respectively). Finally, when calculating the Hessian matrix used for Normal Mode Sampling, we confirm that the energy increases in all directions to check that a given conformer really represents a local minimum (and not a saddle point) of the PES. Structures which fail this test are removed from the database.

#### Quantum chemical calculations

We only run GFN2-xTB calculations if the GFN0-xTB runs finish without any errors. Further, GFN2-xTB calculations which do not converge within 250 iteration steps are discarded. For performing PBE0 calculations with FHI-aims, we randomly sample input structures for which GFN2-xTB successfully converges and discard any calculation which requires more than 1,000 self-consistent field (SCF) iterations to converge, or for which the FHI-aims output reports any other error.

#### Outlier detection

Even though we already filter out disconnected chemical graphs, conformers that do not correspond to the same molecular structure as their parent chemical graph, and calculations for which SCF convergence is problematic, the generation of off-equilibrium structures with normal mode sampling may introduce some conformations which can be problematic when training ML models. In particular, when normal modes are overly “soft” (meaning they are associated with a small force constant), it is possible that conformations with strongly compressed or stretched bonds, corresponding to highly repulsive or dissociated structures, are generated. When training ML models on these inputs, they can hinder convergence, for example due to causing numerical issues. Nonetheless, the data points are still valid calculation results and may provide useful information in some applications. For this reason, instead of completely removing these data points from our dataset, we instead provide a Boolean is_outlier flag, which is True when an entry is detected as potentially problematic according to some filter criteria (see below). We recommend that users skip data points marked as outliers when training or evaluating ML models by default, unless they want to use custom filter criteria or their training pipeline is robust to outliers. Despite our best efforts to mark problematic data as outliers, it is still possible that, e.g., some SCF calculations did not converge to the lowest root, or other issues are present for some data points. Although we did not observe any issues when training ML models on our filtered dataset (see Machine learning models for details), it is still possible that some model architectures may face problems when trained on the QCML dataset. In such cases, we recommend the use of a robust (outlier-resistant) loss function^80^.

Our outlier detection is based on four different criteria:

1. Formation energy: If a structure has a positive formation energy (see Supplementary Section 1 for details how we define formation energy), this means that the constituent atoms at infinite separation would have a lower energy, and the structure thus does not correspond to a stable molecule. Therefore, we mark such entries as outliers.
2. Maximum force: Since potential energy surfaces tend to be fairly smooth, the forces acting on individual atoms in “physical” structures (meaning they are energetically accessible at reasonably low temperatures) tend to be within a narrow numerical range (see Fig. 4b). Extremely large forces typically only occur when atoms are spatially very close to each other, so that the repulsion between the positive charges of the nuclei is the dominant contribution. We flag any structure for which a force acting on an individual atom exceeds $0.5\,{E}_{{\rm{h}}}{a}_{0}^{-1}$ as outlier.

**Fig. 4:**
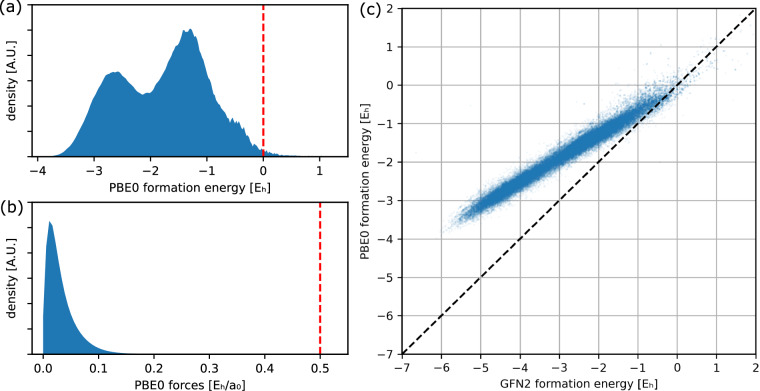
**a**) Distribution of formation energy of the DFT calculations. The dashed vertical line indicates the threshold for the outlier detection of 0.0*E*_h_ (0.9 % of the results are above the threshold). (**b**) Distribution of forces of the DFT calculations. The dashed vertical line indicates the threshold for the outlier detection of $0.5\,{E}_{{\rm{h}}}{a}_{0}^{-1}$ (0.04 % of the results are above the threshold). (**c**) Correlation between GFN2 and PBE0 formation energy for a subsample of 0.2 % of the examples.
3. Minimum normalised inter-atomic distance: Although the maximum force criterion already detects most structures with atoms that are “unphysically” close, it is possible that multiple repulsive interactions cancel out and the net force on an atom is small, despite short inter-atomic distances. Using a normalised distance is motivated by the fact that whether a distance can be considered “unphysically small” or not depends on the size of the atoms involved. The normalised value is computed by dividing the distance by the sum of atomic radii (see Ref. ^81^ for the exact values used for different elements) of the two atoms (a normalised distance of 1.0 roughly corresponds to a typical equilibrium bond length). We flag any structure for which the “normalised distance” between any two atoms is smaller than 0.5 as outlier.
4. Bond order: The conformer generation process already filters out structures for which bond orders deviate strongly from the value expected from graph connectivity (see above). When off-equilibrium structures are generated via normal mode sampling, bond orders naturally fluctuate, so the criterion used for the conformer generation step is typically too strict. Instead, we monitor the bond orders between all atoms which are covalently bonded (according to the chemical graph), and flag a structure as an outlier if any of these bond orders drops below a value of 0.25. Different bond types (i.e., single, aromatic, double, or triple bonds) all share the same threshold. We empirically found that a value below 0.25 reliably indicates dissociation of a bond.

We note that many structures that fail either of the first three checks often also fail the other two, but this is not always the case. In total, roughly 1.5% of entries in our dataset are marked as outliers, with about 0.4% failing at least one of the first three checks and the remaining 1.1% failing the bond order check.

### Data analysis

#### Energy and force distribution

We visualise the distribution of PBE0 formation energies and forces in Fig. 4a and b. For the energy distribution, the bimodal shape stems from the different sampling rates per number of heavy atoms from the normal mode samples for DFT calculations (see Supplementary Figure 1 for a visualisation of the dependency of formation energy on number of heavy atoms). For the distribution of forces, we sample one random force for each molecule. For both plots, the vertical, dashed line indicates the threshold which was applied during outlier detection. 0.9 % of the energies and 0.04 % of the forces are above the cutoff. We note that we consider a result an outlier if any of four criteria is met (see Outlier detection) resulting in approximately 1.5 % outliers overall.

#### Correlation between different levels of theory

For our 33.5M DFT calculations, GFN0 and GFN2 results are available additionally. In Fig. 4c, we compare formation energies computed with PBE0 and GFN2, and observe a high degree of correlation, however with a systematic error. We show that this error can be corrected for when considering the relevant difference of energies for different samples of the same molecule. See Supplementary Section 2.2 for a detailed description of the analysis and its results. We therefore hypothesise that transfer learning approaches taking advantage of our 14.7B GFN2 results will be useful.

### Machine learning models

We leverage our dataset to demonstrate the training of a state-of-the-art machine learning force field and running molecular dynamics simulations with it. In the context of this study, this serves as a verification of the data by applying it for one possible use case. As an example, we choose the SpookyNet^11^ model for its ability of incorporating electronic degrees of freedom (i.e., correct treatment of non-singlet and charged structures). We predict PBE0 formation energy and forces using atomic numbers, positions, and the electronic state (total charge and multiplicity) as input. A detailed description of inputs, outputs, and model and training parameters can be found in Supplementary Section 3.

**Fig. 5:**
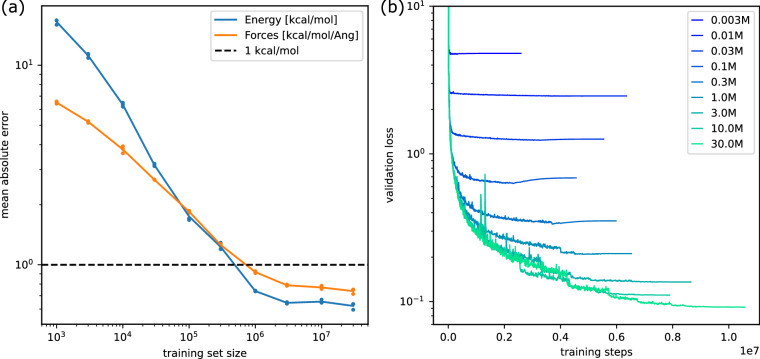
**(a)** Mean absolute error of energy and force predictions on the test set for three different models trained on each given number of examples from the training data. The solid lines indicate the mean over the three replicates per training set size (see also Table 3). **(b)** Average loss (y-axis) on the validation data as a function of training step (x-axis) for models trained on various numbers of examples from the training data.

We partition the DFT calculations of our data randomly into training, validation and test sets. From the fixed training split, we select increasing numbers of up to 30M examples (*N* ∈ [1*K*, 3*K*, 10*K*, 30*K*, …, 30*M*]) while the 10K examples for each of the validation and test split are fixed. The models were trained with the Adam optimiser^82^ and the reduce-on-plateau learning rate schedule (see https://github.com/google-deepmind/optax/blob/main/optax/contrib/_reduce_on_plateau.py). We batch molecules dynamically depending on the maximal number of atoms and edges which results in an average batch size of approximately 21 molecules. Table 3, Fig. 5a, and b summarise the training results. For each training set size and each of the three replicate runs, we select the model with the lowest validation error (early stopping) and report the corresponding test error. The mean absolute errors of the formation energy and forces decrease with increasing amounts of data and saturate between 10M and 30M training examples. Energy and force errors decrease below chemical accuracy with 1M examples. For the models trained on 30M examples, we observe the best validation performance at training step 9.3M, 10.2M and 11.6M, respectively.

**Table 3:** Mean absolute error of energy and force predictions on the test set for different numbers of training examples.

Size of training set	MAE Energy [kcal/mol]	MAE Forces [kcal/mol/Å]
1 000	16.502584 ± 0.470289	6.504683 ± 0.084619
3 000	11.181537 ± 0.270188	5.209448 ± 0.054425
10 000	6.345185 ± 0.144204	3.784762 ± 0.153096
30 000	3.157505 ± 0.044690	2.668150 ± 0.005196
100 000	1.746737 ± 0.097086	1.846259 ± 0.020862
300 000	1.228972 ± 0.052518	1.256822 ± 0.013902
1 000 000	0.737996 ± 0.003321	0.919077 ± 0.009727
3 000 000	0.644416 ± 0.005965	0.789253 ± 0.006055
10 000 000	0.653546 ± 0.013478	0.769623 ± 0.014462
30 000 000	0.621885 ± 0.023161	0.737709 ± 0.020831

The average and standard deviation of three independent runs is reported (see also Fig. 5a).

Although models trained on 1M examples or more achieve energy and force errors below chemical accuracy, recent work^83^ demonstrated that, despite low prediction errors, models may exhibit catastrophic prediction artefacts that lead to the sampling of unphysical structures during molecular dynamics simulations. This can be indicative of insufficient sampling of conformational space in the data that was used to train the model^83^. As a final test for the quality of our data, we therefore use a SpookyNet model trained on 30M examples to perform a 1 ns molecular dynamics simulation of aspirin in the NVE ensemble, using a time step of 0.5 fs. The simulation was initialised with the procedure described in Ref. ^84^, such that the average kinetic energy during the simulation corresponds to a temperature of 300 K. We find that the simulation is stable over the whole trajectory and exhibits no artefacts that would indicate insufficient sampling of conformational space. We chose aspirin as a test system because it contains 21 atoms (13 heavy atoms) and is therefore larger than all structures in the QCML dataset^44^. As such, it also probes whether models trained on the QCML dataset can extrapolate to larger structures.

## Usage Notes

We provide the generated data as Tensorflow dataset (TFDS). Directories consist of dataset/config/version, and dataset names of dataset/config:version. Correspondingly, in Python, tfds.load(name, data_dir=dir), with name = qcml/dft_pbe0_force_field:1.0.0 and dir pointing to the download directory, loads data to train a force field with the PBE0 data.

The per-feature partitions of our dataset can be joined together on-the-fly in an input pipeline using TFDS’s zip function. This however requires disabling file interleaving in order to preserve the order of the elements over different zipped partitions. Each (per-feature) example in each partition contains a hash of the original row key in the field key_hash, which allows for additional verification of the correct order during merging.

We refer to Listing 1 in the supplement, and to the example Python script at the download location for a full example.

## Supplementary information

Supplementary Information

## Data Availability

Starting from SMILES, the generation of 3D structures is performed using OpenBabel^46^ and ASE^59^. We execute semi-empirical calculations with xTB^85^, and DFT calculations with FHI-aims^69^. We use the built-in MBD-NL^77^ dispersion correction of FHI-aims, and the dftd4 software^78^ for the DFT-D4 correction. We use Python wrapper scripts to make these codes compatible with Google Cloud Dataflow and Google Cloud Batch, respectively. All necessary parameters are available in Methods and in the Supplementary Information, and no further custom code was used. Subsequently, we detail the software, their dependencies and version numbers used to execute the steps as described in the corresponding sections above: Atomic Simulation Environment 3.22.1, ChemCoord 2.1.0, DFT-D4 3.5.0, FHI-aims 221103, OpenBabel 3.1.1, Python 3.9.17, libMBD 0.12.6, py-xtb 22.1, xtb 6.6.0.
