# Supplementary material for: The QCML dataset, Quantum chemistry reference data from 33.5M DFT and 14.7B semi-empirical calculations
Source: Sci Data. 2025 Mar 8;12:406. doi: 10.1038/s41597-025-04720-7 (PMC11890765; doi:10.1038/s41597-025-04720-7)
Supplement: Supplementary file 1 — Supplementary Information [file 41597_2025_4720_MOESM1_ESM.pdf]

Supplementary Information

Stefan Ganscha[1,†,*], Oliver T. Unke[1,†,*], Daniel Ahlin[1], Hartmut Maennel[1], Sergii Kashubin[1], and Klaus-Robert Müller[1,2,3,4,*]

[1]Google DeepMind
[2]Machine Learning Group, TU Berlin and BIFOLD, Berlin, Germany
[3]Department of Artificial Intelligence, Korea University, Seoul, Korea
[4]Max Planck Institute for Informatics, Saarbrücken, Germany
[*]corresponding author(s): Stefan Ganscha (ganscha@google.com), Oliver T. Unke (oliverunke@google.com), Klaus-Robert Müller (klausrobert@google.com, klaus-robert.mueller@tu-berlin.de)
[†]these authors contributed equally to this work

Supplementary Section 1 Additional information about properties, metadata, and conventions

Supplementary Section 1.1 Metadata fields summary

In addition to the properties listed in Table 2, the QCML database also includes metadata fields containing additional information about individual entries, see Supplementary Table 1 for a summary. The combination of the fields `smiles`, `conformation_parent_seq` and `conformation_seq` is a unique identifier of an example (and conformation, respectively). Additionally, it can be used to find related entries. For example, all entries with the same `conformation_parent_id` are derived from the same chemical graph, and if `conformation_parent_seq` of two entries are equal, they are normal mode samples of the same conformer. In addition, since our implementation of the normal mode sampling procedure (see main text) samples structures at increasingly larger temperatures in order, structures with `conformation_seq = 0` always correspond to a temperature of 0 K, i.e. equilibrium structures (at the GFN0-xTB level of theory).

**key**	**type**	**description**
`smiles`	`str`	SMILES string of the parent chemical graph
`chemical_formula`	`str`	Chemical (empirical) formula
`molecular_weight`	`float`	Molecular weight in atomic mass units
`num_atoms`	`int`	Number of atoms in the structure
`num_heavy_atoms`	`int`	Number of heavy (non-hydrogen) atoms in the structure
`pbe0_has_equal_a_b_electrons`	`bool`	Whether `{a|b}` fields (for α and β electrons) are identical
`pbe0_num_scf_iterations`	`int`	Number of SCF iterations until convergence
`conformation_seq`	`int`	Metadata identifier for conformation
`conformation_parent_seq`	`int`	Metadata identifier for conformation
`is_outlier`	`bool`	Outlier according to the described procedure

Supplementary Table 1. Metadata fields in the QCML database.

Supplementary Section 1.2 Matrix and grid quantities

The QCML database contains some properties related to the wave function (ansatz) (see Table 2). In some cases, two versions of a property may exist, one for the α and one for the β electrons (denoted by suffixes `*_a` and `*_b`). However, for closed-shell calculations, these entries are identical, so storing both values would be wasteful. For this reason, we only store the `*_a` properties in these cases and set the metadata field `pbe0_has_equal_a_b_electrons` (see Supplementary Table 1) to `True` to indicate that the missing `*_b` field is identical to the `*_a` field.

Many *ab initio* codes treat closed-shell systems in a special way for computational efficiency, by essentially only instantiating orbitals for α electrons, but allowing double occupations. For consistency between results for closed-shell and open-shell calculations, we manually convert results for closed-shell calculations so that the results are identical to a calculation that does not treat closed-shell systems in a special manner – for example, by halving orbital occupations or the magnitude of entries of the density matrix.

Supplementary Section 1.3 Multipoles

There exist different conventions for defining multipole moments in the literature. The simplest definition uses so-called raw/primitive moments, which are computed as (definition 1)

$$\begin{aligned}
M &= \int \rho(\mathbf{r})\,\mathrm{d}\mathbf{r} \\
D_\alpha &= \int \rho(\mathbf{r}) \cdot r_\alpha\,\mathrm{d}\mathbf{r} \\
Q_{\alpha\beta} &= \int \rho(\mathbf{r}) \cdot r_\alpha r_\beta\,\mathrm{d}\mathbf{r} \\
O_{\alpha\beta\gamma} &= \int \rho(\mathbf{r}) \cdot r_\alpha r_\beta r_\gamma\,\mathrm{d}\mathbf{r} \\
&\vdots
\end{aligned} \tag{1}$$

where $\rho(\mathbf{r})$ is the charge density at position $\mathbf{r} = [r_x\ r_y\ r_z]^\top$, and α, β, and γ can stand for either x, y, or z. Here, M is the monopole moment (order 0), and D_α, $Q_{\alpha\beta}$, and $O_{\alpha\beta\gamma}$ are the entries of the dipole (order 1), quadrupole (order 2) and octupole (order 3) moments, respectively. Starting from order 2, these moments are not "rotationally pure" (i.e., they are not irreducible representations, but contain "contaminations" from lower orders). It is possible to define traceless variants of the multipole moments, which are irreducible. They can either be calculated directly, or derived from the primitive moments using a detracing operator.[1] This leads to multipole moments (definition 2)

$$\begin{aligned}
Q_{\alpha\beta} &= \int \rho(\mathbf{r}) \cdot \left(r_\alpha r_\beta - \frac{1}{3}\|\mathbf{r}\|^2 \delta_{\alpha\beta} \right) \mathrm{d}\mathbf{r} \\
O_{\alpha\beta\gamma} &= \int \rho(\mathbf{r}) \cdot \left(r_\alpha r_\beta r_\gamma - \frac{1}{5}\|\mathbf{r}\|^2 \left(r_\alpha \delta_{\beta\gamma} + r_\beta \delta_{\alpha\gamma} + r_\gamma \delta_{\alpha\beta} \right) \right) \mathrm{d}\mathbf{r} \\
&\vdots
\end{aligned} \tag{2}$$

where δ is the Kronecker delta. Note that there exist various definitions for traceless multipole moments in the literature (essentially different "normalisations" are used), e.g., another common definition for the traceless quadrupole and octupole are (definition 3)

$$\begin{aligned}
Q_{\alpha\beta} &= \int \rho(\mathbf{r}) \cdot \left(3 r_\alpha r_\beta - \|\mathbf{r}\|^2 \delta_{\alpha\beta} \right) \mathrm{d}\mathbf{r} \\
O_{\alpha\beta\gamma} &= \int \rho(\mathbf{r}) \cdot \left(15 r_\alpha r_\beta r_\gamma - 3\|\mathbf{r}\|^2 \left(r_\alpha \delta_{\beta\gamma} + r_\beta \delta_{\alpha\gamma} + r_\gamma \delta_{\alpha\beta} \right) \right) \mathrm{d}\mathbf{r} \\
&\vdots
\end{aligned} \tag{3}$$

This normalisation is often convenient, because it allows to express the electrostatic potential as

$$\Phi(\mathbf{r}) = \frac{M}{\|\mathbf{r}\|} + \sum_\alpha \frac{D_\alpha}{\|\mathbf{r}\|^3} r_\alpha + \sum_\alpha \sum_\beta \frac{Q_\alpha}{\|\mathbf{r}\|^5} r_\alpha r_\beta + \dots \tag{4}$$

(the sums run over the components x, y, and z). The conversion from definition 2 to definition 3 can be achieved by multiplying the multipole components by a factor of $(2\ell-1)!!$, where ℓ is the order of the multipole and '!!' is the double factorial. All of these definitions have in common that the multipole moment of order ℓ has 3^ℓ entries, of which, however, only $\frac{(\ell+1)(\ell+2)}{2}$ are unique (due to symmetry). In principle, the traceless moments can be stored with one fewer value (because their trace is zero), but there is an even better way: Spherical multipole moments, which require only $2\ell+1$ values, but contain the same information. They are defined as (definition 4)

$$\Omega_\ell^m = \int \rho(\mathbf{r}) \cdot R_\ell^m(\mathbf{r})\,\mathrm{d}\mathbf{r} \tag{5}$$

where $R_\ell^m(\mathbf{r})$ is a regular solid harmonic and Ω is a placeholder that stands for any multipole moment. Here, ℓ is the multipole order and m runs from $-\ell$ to ℓ. All multipole moments in the QCML database are stored as spherical multipole moments following the definition in (5) and the entries are ordered from $m = \ell, -\ell, \ell-1, -\ell+1, \dots, 0$. This seemingly arbitrary order is chosen so that the entries of the dipole moment coincide with the typical (x, y, z)-order used in the other definitions (see above), so that dipole moments are equivalent to definitions 1-3.

Supplementary Section 1.4 Formation energy

Raw energy outputs from *ab initio* calculations often have large absolute values. When training ML models, which is typically done with single (or even lower) precision floating point numbers, the large absolute values can cause numerical issues (due to loss of precision), which may lead to sub-optimal models. For this reason, it is often convenient to instead use formation energies when training ML models, which typically have smaller absolute values. The formation energy is obtained by subtracting the energy of all constituent atoms at infinite separation (“free atoms”) from the raw energy output (note that subtracting arbitrary constants from the energy does not change the underlying physics). The free atom reference values used to compute formation energies in the QCML database can be found in Supplementary Table 2. We recommend that practitioners train models on formation energies (instead of raw energies). If necessary, energy predictions from models that were trained on formation energies can be trivially converted to raw energies by re-addition of the respective constants.

Z	GFN0-xTB	GFN2-xTB	PBE0/tight
1	−0.438138811313	−0.393482763936	−0.4999857166405113
2	−1.540036500274	−1.743126632946	−2.8931179195666323
3	−0.25714125125	−0.180071686575	−7.46273130932408
4	−0.721128011821	−0.569105981616	−14.633885134967791
5	−1.111060684505	−0.950622467488	−24.616585670571556
6	−1.659128780731	−1.622879464565	−37.81875063685348
7	−2.522819050697	−2.530676419942	−54.58427980346498
8	−3.058521580106	−3.7657928037	−75.08703715456889
9	−3.779182577845	−4.617525817562	−99.80800992103507
10	−4.557378331611	−5.932215052758	−129.08936802709763
11	−0.294614136152	−0.167096749822	−162.509867671
12	−0.654110158939	−0.465974663792	−200.4492628250367
13	−1.041642586289	−0.903514464803	−242.9313205013714
14	−1.53918753577	−1.426947780447	−290.1990620477859
15	−2.135674407723	−2.231644397682	−342.42127720788295
16	−3.125980204378	−3.144642723727	−399.6742295174213
17	−4.203158677511	−4.480710988285	−462.2183432820921
18	−4.774866930193	−4.27904326759	−530.2246874943389
19	−0.245931237521	−0.165752239061	−603.3529526409765
20	−0.591899095559	−0.371646352489	−681.9031353517017
21	−0.739813716318	−0.59101486273	−766.0094116430901
22	−1.063159436642	−1.003162644968	−855.9774833559461
23	−1.375298292653	−1.371705994803	−951.9925136068362
24	−1.606743409778	−1.42357461514	−1054.2188469854264
25	−2.122063294239	−2.220421154196	−1162.6251958858654
26	−2.580354170542	−2.532937477212	−1277.480439914856
27	−2.988212876727	−3.132713966722	−1399.01286844174
28	−3.443662699558	−4.156386813541	−1527.3620116218892
29	−4.485406875678	−3.748006130985	−1662.6360171135063
30	−0.812198583769	−0.527521402296	−1805.0185044630823
31	−0.995504387234	−1.079297689038	−1954.3125384262237
32	−1.701159153235	−1.656434261511	−2110.7549593414697
33	−2.238417395442	−2.102842997329	−2274.4573449272452
34	−3.188622557822	−3.116807903904	−2445.454608787767
35	−3.599724292608	−4.046525224558	−2623.9522516534653
36	−4.240238664367	−4.271855540848	−2810.0746311240428
37	−0.24471611378	−0.159998948675	−3003.5294734508125
38	−0.467104286557	−0.462430853001	−3204.5832947894182
39	−0.70322020966	−0.778188319378	−3413.2951654665567
40	−1.033074110279	−0.958632129605	−3629.9351943326074
41	−1.375892646817	−1.352751575577	−3854.6810399669666
42	−1.532980883677	−1.580331244477	−4087.61703939783
43	−2.017985552705	−2.133572175519	−4328.782950508711
44	−2.629939764863	−2.585395446455	−4578.421512450055
45	−3.226521062367	−3.423379913548	−4836.674864745159
46	−3.573963262851	−3.948989873266	−5103.804022705202
47	−4.264029482027	−3.821738210271	−5379.567778210477
48	−0.882588319406	−0.533037255301	−5664.357522325995
49	−1.123411342121	−1.124123632214	−5957.998607776462
50	−2.162283099221	−2.009268322783	−6260.7322158909255
51	−2.42547230061	−2.160600494682	−6572.679459595302
52	−3.061725731598	−3.005462671221	−6893.899281116733
53	−3.452939092492	−3.777816116714	−7224.593807216719
54	−3.949988227446	−3.88358849819	−7564.897946935262
55	−0.231283111405	−0.148529962461	−7914.618376213088

56	−0.417443617042	−0.433642020732	−8274.018647543899
57	−0.75741164818	−0.787828695989	−8644.356283089124
58	−0.838413780044	−0.695932234438	−9022.719691377077
59	−0.830848369476	−0.685699752722	−9412.304317317776
60	−0.823282955234	−0.675467234256	−9812.733623074671
61	−0.815717540993	−0.66523475254	−10223.959035470503
62	−0.808152126751	−0.655002234074	−10646.160908789849
63	−0.800586716184	−0.644769752358	−11079.523153772532
64	−0.793021301942	−0.634537270641	−11524.170045254037
65	−0.785455891375	−0.624304752176	−11980.1372554151
66	−0.777890477133	−0.614072270459	−12447.91726607854
67	−0.770325062891	−0.603839788743	−12927.641007633441
68	−0.762759648649	−0.593607270278	−13419.499880526915
69	−0.755194234408	−0.583374788561	−13923.704752181095
70	−0.747628820166	−0.573142233347	−14440.47104140629
71	−0.740063409599	−0.562909788379	−14969.934870697594
72	−0.988225106902	−0.977305306258	−15512.207108420227
73	−1.430310530996	−1.391398965191	−16067.493075934339
74	−1.744021004914	−1.856554707053	−16636.009874752825
75	−2.112721877052	−2.663692930985	−17217.983148242605
76	−2.508513648775	−2.740576595531	−17813.536948696015
77	−3.287377799242	−3.310461809808	−18423.032971689845
78	−3.765724986497	−4.066034374829	−19046.681751999644
79	−4.796354278188	−3.802619448068	−19684.814481937072
80	−0.894444106953	−0.848032246708	−20337.64746578749
81	−1.665317506504	−1.436870998229	−21005.18286079211
82	−2.219103830586	−2.201212686894	−21687.832194337443
83	−2.360919853288	−2.263025123009	−22385.879580271372
84	−3.237949381779	−2.731153444172	−23099.562332173726
85	−3.136841198754	−2.998728945625	−23829.264304097684
86	−3.686505836277	−3.857886535621	−24575.317556488535

Supplementary Table 2. Free atom reference energies (in E_h) for elements from H ($Z = 1$) to Rn ($Z = 86$) (used to calculate the fields `{gfn0|gfn2|pbe0}_formation_energy` (for the different levels of theory).

Supplementary Section 2 Additional figures and tables

Supplementary Section 2.1 Formation energy distribution per number of heavy atoms

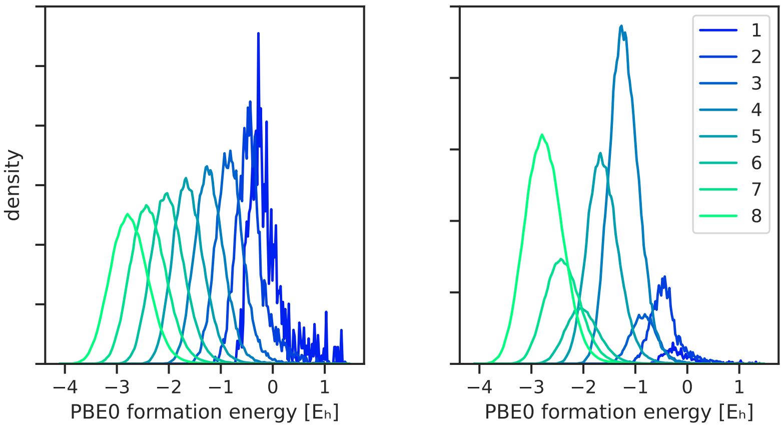

Supplementary Figure 1. Histogram of PBE0 formation energies per number of heavy atoms. (Left) Normalised per number of heavy atoms individually. (Right) Normalised with total count.

Supplementary Section 2.2 Comparing DFT and xTB energies

In Figure 4c we compared not directly the energy outputs of the DFT-PBE0 computation with the GFN2-xTB computation, but only energy differences between conformations of the same molecule. The reason is that absolute energies are somewhat arbitrary for different molecules, and the difference between conventions about the energy are on a larger scale (1-10 for xTB, 1000-10000 for PBE0, see Supplementary Table 2) than the error in the prediction for energy difference as in Figure 4c (of

order 0.1). Therefore, comparing directly the outputs of energies would give results that make the comparison between different methods either look completely unusable (if we do not subtract the different constants from Supplementary Table 2 for each atom) or look incredibly precise (because after correcting with the right offsets per element we would intuitively compare the deviation from the diagonal in the order of 0.1 with the scale of the energies in the order of 1000-10000). So instead of the raw energies we plot in Supplementary Figure 2 the predicted formation energies, which are more comparable between methods.

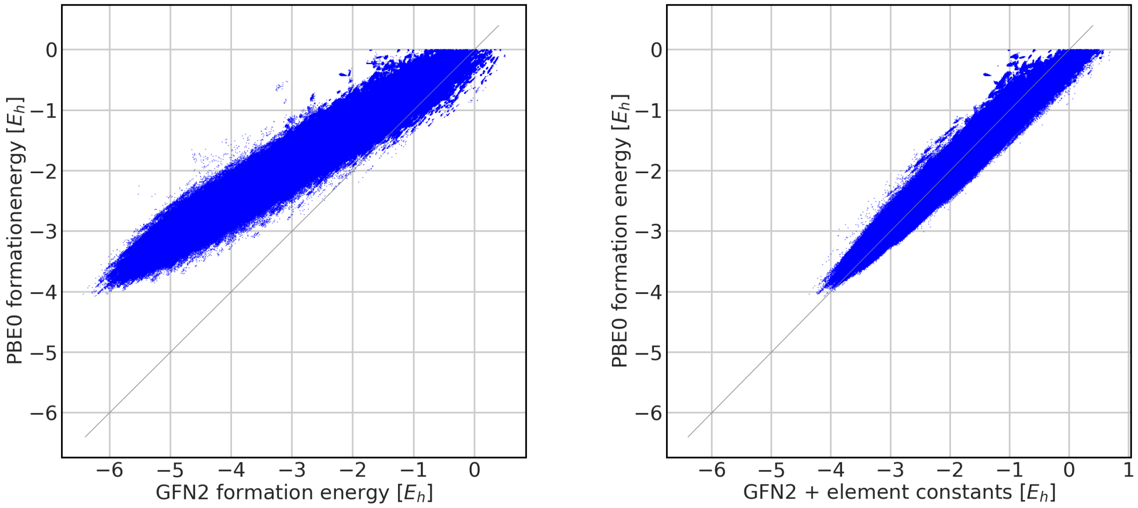

Supplementary Figure 2. Direct comparison of formation energies. Left is the correlation plot of the original data, right is a version that corrects for slightly different versions of formation energies by subtracting constants per element. The data are restricted to `is_outlier=False`, which enforces the condition that PBE0 formation energies are negative, resulting at the cutoff at the top.

As there are also slightly different versions of computing “formation energies”, we again get slightly different constants per element. Correcting for them gives the plot on the right hand side of Supplementary Figure 2, this corresponds to what we could use if we wanted to predict PBE0 formation energies in cases where we only have GFN2 formation energies.

However, in most cases (in particular for accuracy of simulations of the dynamics), we do not compare formation energies of different molecules, but are only interested in the energies assigned to different conformations of the same molecule, up to an additive constant, i.e. we are interested in energy differences between different conformations of the same molecule. To measure the accuracy with which GFN2 would predict such energy differences, we constructed lists of conformations of the same molecule (i.e. records with the same SMILES string) and used energy differences between the first conformation in these lists and all other conformations. Plotting these energy differences for both methods gives Figure 4c, which we repeat on the left side of Supplementary Figure 3.

This plot gives over 23 million points, so it outlines also rare outcomes. To get a better estimate of the distribution of errors, we also give them as a histogram, see Supplementary Figure 4.

The restriction to `is_outlier=False` in Figure 4c is used since most users will probably only use these data, and for outliers the formation energy (differences) of GFN2 are less useful for predicting the formation energy (differences) of PBE0 – in particular, when the distance between atoms is too small, the two methods give different estimates about the (large) repulsion, and the corresponding figure for “outlier” conformations looks quite different, see the right hand side of Supplementary Figure 3.

Supplementary Section 2.3 Sampling

Supplementary Section 3 Additional information about MLFF training and parameters.

The hyperparameters used to train SpookyNet model are the same as in the SpookyNet paper, except the following:

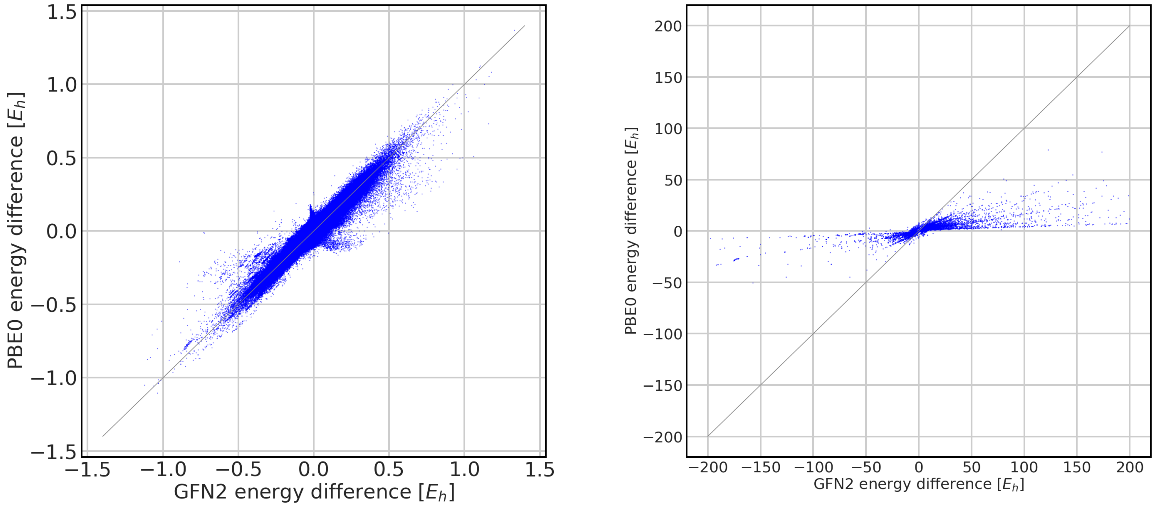

Supplementary Figure 3. Comparison of energy differences given by different methods, left: only `is_outliers=False`, right: only `is_outliers=True`.

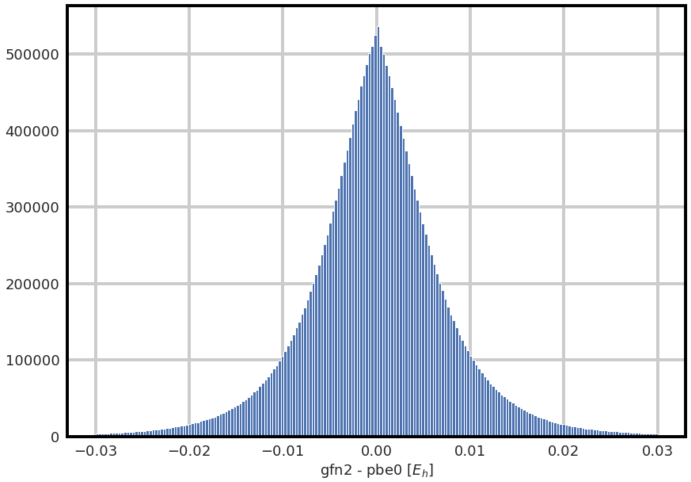

Supplementary Figure 4. Histogram of difference between energy differences in GFN2 vs. PBE0 models.

- Number of interaction modules is 4 (SpookyNet paper uses 6).
- Number of features is 256 (SpookyNet paper uses 128).
- Number of basis functions is 64 (SpookyNet paper uses 16)
- Weights are initialised with uniform orthogonal matrices (SpookyNet paper uses Glorot initialisation[2])
- The dispersion correction and explicit electrostatics contributions are not used
- The optimiser used is Adam with initial learning rate 10^{-4} (SpookyNet paper uses AMSGrad with 10^{-3})
- The exponential moving average of parameters is not used

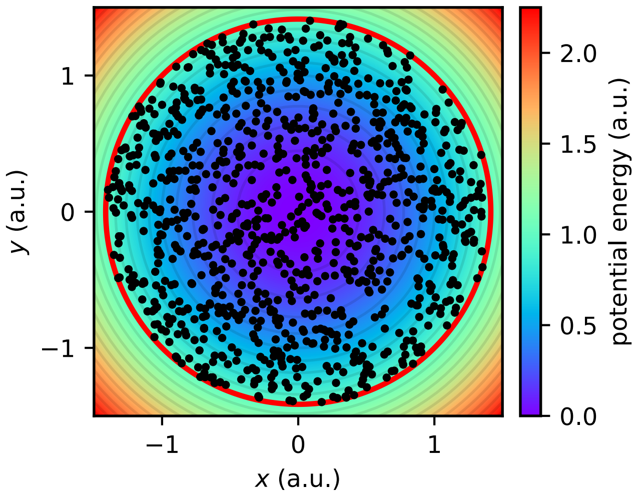

Supplementary Figure 5. Normal mode sampling (see main text) of the harmonic potential $V(x,y) = \frac{x^2}{2} + \frac{y^2}{2}$ with 1 000 energies uniformly selected between 0 and 1. The contour line for $V(x,y) = 1$ is highlighted in red to show that samples (black points) are evenly distributed in the energetically accessible region of the potential energy surface (PES).

maximum valency	lowest normal valencies	elements
1	1	F, Cl, Br, I
2	2	O
3	3	B
4	4	C
5	3, 5	N, P
6	2, 4, 6	S

Supplementary Table 3. Overview of valency information for elements in the SMILES organic subset. If the explicit bonds of an element in the organic subset lead to a valency different from its lowest normal valencies (but lower than its maximum valency), the SMILES standard assumes implicit additional bonds to H atoms until the next valid normal valency is reached (unless specified otherwise). For example, a C atom that has two explicit bonds is assumed to be bonded to two additional H atoms implicitly.

- The reduce-on-plateau learning rate decay scheme is the same but the training is stopped when the learning rate drops below 10^{-9} (SpookyNet paper stops after 10^{-5}) and the evaluation happens every 10^4 steps (SpookyNet paper evaluates every 10^3 steps)
- The dipole moment loss is not used
- The forces loss weight is set to 100 in atomic units (52.91772105638411 in practice as forces are computed in eV/Å) as was for MD17 dataset in the SpookyNet paper.
- The gradients are clipped by global norm with maximal norm of 1000[3]

N_{heavy}	N_{samples}
≤ 5	1 000
6	500
7	200
≥ 8	100

Supplementary Table 4. Number of normal mode samples N_{samples} per conformer depending on the number of heavy atoms N_{heavy}. We generate more samples for smaller structures because they have fewer conformers on average.

```python
import tensorflow as tf
import tensorflow_datasets as tfds

read_config = tfds.ReadConfig(
    interleave_cycle_length=1,
)
data_dir = '/downloaded/tfds/'
training_split='train[:90%]'

tfds_name_0 = 'qcml/dft_atomic_numbers:1.0.0'
train_ds_0 = tfds.load(
    tfds_name_0, split=training_split, data_dir=data_dir, read_config=read_config
)

tfds_name_1 = 'qcml/dft_positions:1.0.0'
train_ds_1 = tfds.load(
    tfds_name_1, split=training_split, data_dir=data_dir, read_config=read_config
)

ds = tf.data.Dataset.zip([train_ds_0, train_ds_1])
```

Listing 1. Example of loading two Tensorflow datasets and zipping them together.

Supplementary Section 4 Additional information about data records and usage

In 1 we exemplify the usage of a TFDS, particularly the usage of the `zip` function to merge two per-feature partitions. Note the `read_config`, which is necessary to keep the record order when reading from different TFDSs in parallel. For a single dataset, without merging, the code reduces to a single `tfds.load` statement.

References

1. Applequist, J. Traceless cartesian tensor forms for spherical harmonic functions: New theorems and applications to electrostatics of dielectric media. *J. Phys. A: Math. Gen.* **22**, 4303 (1989).
2. Glorot, X. & Bengio, Y. Understanding the difficulty of training deep feedforward neural networks. In Teh, Y. W. & Titterington, M. (eds.) *Proceedings of the Thirteenth International Conference on Artificial Intelligence and Statistics*, vol. 9 of *Proceedings of Machine Learning Research*, 249–256 (PMLR, Chia Laguna Resort, Sardinia, Italy, 2010).
3. Pascanu, R., Mikolov, T. & Bengio, Y. On the difficulty of training recurrent neural networks (2013). 1211.5063.
